# Ultra-flexible Piezoelectric Devices Integrated with Heart to Harvest the Biomechanical Energy

**DOI:** 10.1038/srep16065

**Published:** 2015-11-05

**Authors:** Bingwei Lu, Ying Chen, Dapeng Ou, Hang Chen, Liwei Diao, Wei Zhang, Jun Zheng, Weiguo Ma, Lizhong Sun, Xue Feng

**Affiliations:** 1 AML, Department of Engineering Mechanics, Tsinghua University, Beijing, 100084, China; 2 Center for Mechanics and Materials, Tsinghua University, Beijing, 100084, China; 3 Beijing Anzhen Hospital, Capital Medical University, Beijing, 100029, China

## Abstract

Power supply for medical implantable devices (i.e. pacemaker) always challenges not only the surgery but also the battery technology. Here, we report a strategy for energy harvesting from the heart motion by using ultra-flexible piezoelectric device based on lead zirconate titanate (PZT) ceramics that has most excellent piezoelectricity in commercial materials, without any burden or damage to hearts. Experimental swine are selected for *in vivo* test with different settings, i.e. opened chest, close chest and awake from anesthesia, to simulate the scenario of application in body due to their hearts similar to human. The results show the peak-to-peak voltage can reach as high as 3 V when the ultra-flexible piezoelectric device is fixed from left ventricular apex to right ventricle. This demonstrates the possibility and feasibility of fully using the biomechanical energy from heart motion in human body for sustainably driving implantable devices.

Performance of implantable devices heavily relies on power supply. With the rapid development of nanotechnology, implantable devices are fabricated and produced smaller and smaller but have to face the serious challenge of the power supply. In spite of lots of improvement, battery’s power supply is far behind the requirement of implantable devices. On the other hand, the limited service time is always the Achilles’ heel, which exposes the patients to potentially deadly risks, because of power dissipation and the need for replacement^1^. Multiple wireless power methods are developed to overcome the drawbacks of the battery, yet new problems like power efficiency and temperature rise ensue^2^,^3^,^4^.

Mechanical energy exists in the human body is prevalent during all life, such as motion of the heart, contraction/relaxation of the diaphragm and lungs, building a self-powered implantable system by energy harvesting from body or organ is well promising. Recent advances in nanotechnology and flexible electronics have allowed the creation of such devices. Many researches has been done in this area, including ZnO nanowires integrated with flexible substrate converted biomechanical energy of human body activity into electric energy by using both piezoelectric and triboelectric effects^5^,^6^,^7^. Other efforts were made to study fabrication of such flexible energy harvester based on PZT, PVDF and other piezoelectric materials^8^,^9^,^10^,^11^ and demonstrate the possible energy harvesting from walking or running^12^,^13^,^14^,^15^. These biomechanical energy harvesting devices have achieved considerable electric energy taking advantage of body motions and are promising in applications like potable and wearable electronics^16^,^17^.

Biomechanical energy harvesting is a promising strategy to solve the power supply problems in medical implants, among which scavenge energy from heart motion is of special importance for its regularity, life-long lasting and relative insusceptibility to external environment. Piezoelectric material is commonly used to convert mechanical energy into electric energy. The efficient energy conversion from heart motion requires excellent sensitivity and high piezoelectric coefficients of the piezoelectric material, since the mechanical energy derived from cardiac motion is weak. Meanwhile, considering the softness of biological tissue as well as the healthy conditions of the patient with medical implants, the energy harvester integrated with heart should not cause any damage or extra burden to the patient. Therefore, high sensitivity and excellent flexibility are necessary for the energy harvesters. The most promising application of such flexible energy harvester is to supply the power for pacemaker aiming at eliminating the need for battery replacement and reducing the related surgical risks. Recently conformal piezoelectric energy harvesting and storage based on PZT has been reported with theoretical models and *in vivo* evaluation on bovine and ovine organs^18^. By converting motions of internal organs like the heart, lungs and diaphragm the piezoelectric energy harvester yields significant electrical power for practical use of medical implants. However, these aforementioned tests have inherent limitations. First, bovine and ovine are not similar to human being with regard to the cardiac anatomy. Second, all these *in vivo* tests are performed in anesthetized animals, which differ considerably from the conscious animals. Thus, the tests remain certain difference from the expected scenario of the biomechanical energy harvester.

In this study, we develop an ultra-flexible energy harvester (UFEH) based on PZT, which can be integrated with the heart to harvest the mechanical energy derived from cardiac motions. These ultra-flexible piezoelectric devices, composed of brittle piezoelectric film with high piezoelectric coefficients and extremely soft substrate, are designed and fabricated via transfer printing technology to achieve excellent flexibility. Experimental model is set up in swine, whose heart anatomy is very close to human beings, for *in vivo* test to demonstrate the feasibility and possible applications of such devices in biomechanical energy harvesting for medical implants (e.g. pacemaker). *In vivo* energy harvesting is done while the swine chest is being opened and closed, as well as when the swine resumes conscious after recovery from anesthesia. In addition, the effects of different manners of suture fixation, mounting places and orientations for achieving a relatively high output voltage are also investigated.

## Results

In order to harvest the biomechanical energy of heart motion, piezoelectric PZT (Pb(Zr_0.52_Ti_0.48_)O_3_), the most efficient and sensitive piezoelectric material^19^, is selected as the energy conversion material. However, it is brittle and cannot be subjected to large deformation with respect to the heart motion. Therefore, fabrication of such flexible PZT-based energy harvester utilized not only traditional lithographic methods but also transfer-printing technology developed especially for flexible electronics to overcome PZT ceramic’s brittleness and fragility^20^,^21^,^22^,^23^.

**Figure 1:**
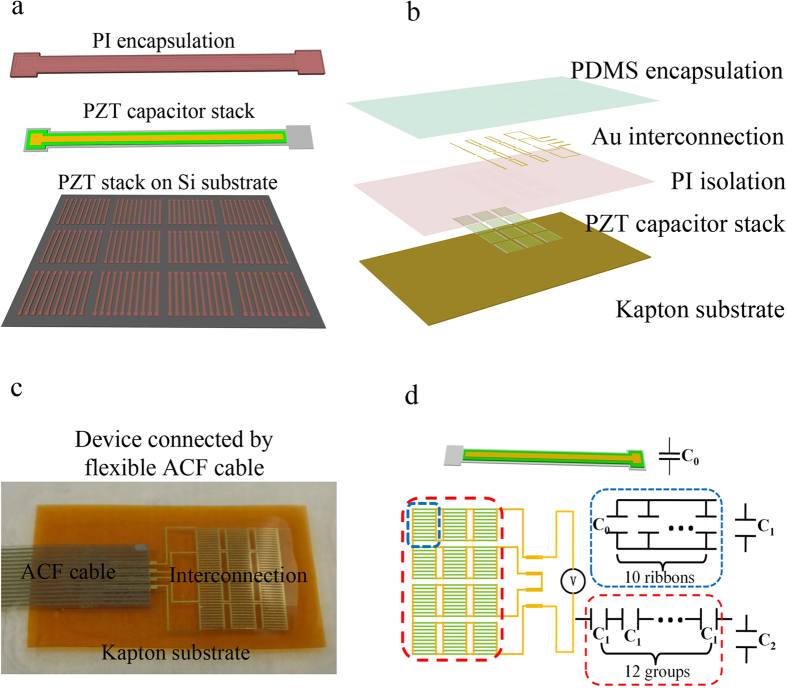
Sketch of fabrication. (**a**) PZT capacitor stacks Fabricated on Si wafer and encapsulate with PI. (**b**) Interconnection and encapsulation manufactured on flexible substrate. (**c**) Device connected with flexible ACF cable. (**d**) Electrical connecting sketch of device.

Commercial PZT wafer (MEMS), with the structure of PZT(500 nm)/Ti(20 nm)/Pt(300 nm)/SiO2(600 nm)/Si(500 um), is fabricated into array of nanoribbons for PZT stacks by lithography and wet etching. Before transfer-printing a layer of polyimide (PI, POME) is spin-coated on the wafer and patterned to encapsulate the PZT stack in order to protect them from destruction in the transfer-printing process as shown in Fig. 1a. Custom-made PDMS stamps are used to pick up the PZT stacks from the silicon substrate after etching of the sacrificial layer, and print them onto the commercially available polyimide thin film (Kapton) with the thickness of 75 *μm* as soft substrate. Transfer-printing is the key step among the fabrication process, which endows the rigid functional material with flexibility and determines the reliability of the final devices. Main factors that decide the success rate are proper contact pressure which guarantees uniform contact strength between the stamp and the nano-/micro- features, and kinetically peeling velocity which determines the adhesion strengths of the interfaces^20^,^21^,^22^. Before the deposition and patterning of the Au interconnection by E-beam and lithography respectively, a layer of polyimide is spin-coated to further isolate the top and bottom electrode from each other. Each ribbon is perforated by RIE (Reactive Ion Etching) at both ends properly ensuring individual electrode’s integration with Au interconnection to form the entire circuit for signal output. Figure 1b illustrates the layered structure of the whole UFEH after successful printing and integration with PDMS encapsulation. Here, PDMS encapsulation enhances the biocompatibility of the device, because PDMS is a biocompatible and popular supporting material in medical experiments. Finally, UFEH is connected with flexible ACF cable (Elform) by clamping the two at 190 °C for 10 min. The finished product is shown in Fig. 1c.

The basic element of UFEH is the PZT-based nano-capacitor, which are connected by Au interconnection to form the integrated device. The thickness of PZT film varies from 400 nm to 600 nm in the proposed devices. Pt and Au are deposited on PZT acting as bottom and top electrode, respectively. The ultra-flexible PZT energy harvester is consist of 12 groups of PZT ribbons (each ribbon is 2000 *μm* long and 100 *μm* wide), which are connected in series for the sake of magnifying the total output voltage. Within each group there are 10 PZT ribbons connected in parallel in order to increase electric charge quantity in each group generated by piezoelectric effect of the PZT ribbon as shown in Fig. 1d, in which the green lines stand for the PZT ribbons, and the golden stands for Au interconnection. The capacitance of each ribbon denoted as *C*_0_, which gives *C*_1_, the capacitance of each group (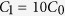), and that of the whole device, which is 
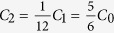
. Theoretical analysis gives the output voltage denoted as *V*, determined by the deformation characterized by the membrane strain of the PZT nanoribbon denoted as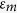, i.e.^18^,


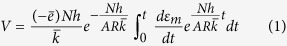


where *N* is the number of PZT ribbon groups, *h* the thickness of PZT ribbons, *A* the total area of PZT ribbons in a single group, *R* the resistance of the voltmeter, and 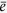, 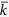 is the effective piezoelectric constant and effective dielectric constant respectively.

**Figure 2:**
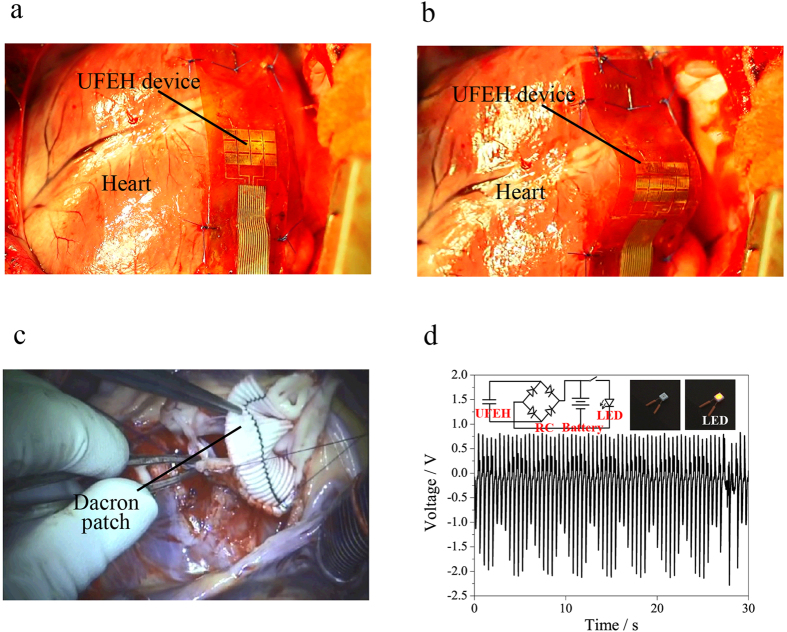
*In vivo* testing. (**a**) Image of device when the heart relaxes in diastoles. (**b**) Image of device when the heart contracts in systole. (**c**) Image of Dacron patch, which is widely used in cardiovascular operation. (**d**) Output voltage measured by AD/DA card.

Experimental swine raised especially for cardiovascular research is chosen to conduct the *in vivo* test of the biomechanical energy harvesting by UFEH with the permission from the Ethics Committee. We succeed in integrating the UFEH with the swine heart by carefully suturing the corners of the device to the properly selected segments on the epicardium as shown in Fig. 2. Figure 2a,b show the UFEH working status of flat at cardiac relaxation (e.g. diastole) and bulge at contraction (e.g. systole), respectively. As we observed in the experiment the existence of the flexible energy harvester does not affect the normal physiological activity of the swine, which is similar to that of Dacron patch widely used in all kinds of cardiac surgical procedures as shown in Fig. 2(c)^24^. Actually, the bending stiffness of UFEH is much smaller than that of patch. The detailed analysis and comparison about the bending stiffness are given in Experimental Section. As the UFEH brings no extra mechanical burden to heart, the device fixed on the epicardium bends and flattens as the heart contracts in systole and relaxes in diastoles rhythmically in cardiac cycles, during which the piezoelectric effect of PZT converts the biomechanical energy into electricity and output by a flexible ACF cable. And the video of swine heart motion with UFEH can be found in the Supplementary Information (Open chest setting). AD/DA card (Art-control USB2815) is used to capture the signal generated during energy harvesting and to transmit the data to a computer. Figure 2d shows the output voltage, measured immediately after chest opened, is periodic pulse synchronized with cardiac beats. The peak-to-peak voltage can reach as high as 3 V most of the time, but the amplitude is non-uniform due to the unstable heart motion just after chest opened. When the rectifying circuits and battery chip are connected to the output voltage, the energy is stored in battery and then the commercial LED is illuminated for demonstration, shown in insets in Fig. 2d, and the video is also available in the Supplementary Information (LED illuminated). So far the feasibility of UFEH to convert biomechanical energy into electricity safely and effectively has been preliminarily demonstrated both medically and electrically.

**Figure 3:**
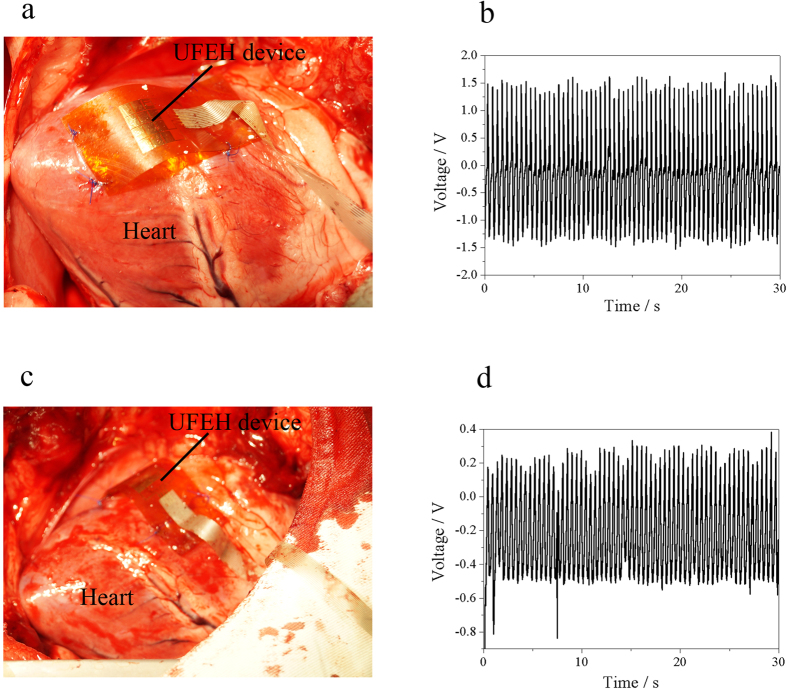
Comparison of output voltage from different locations. (**a**) Image of UFEH mounted between left ventricular apex and right ventricle, which belongs to segment 13. (**b**) Voltage acquired in location in (**a**). (**c**) Image of UFEH mounted between the anterior atrioventricular groove and the right ventricular outflow tract. (**d**) Voltage acquired in location in (**c**).

In fact, the output voltage of UFEH is related to the deformation of the heart due to piezoelectric effect. From the physiological point, the heart deformation is complicated and hybrid with tension, contraction, bending and torsion in a cardiac period, which results from the fact that different parts of the cardiac muscle contract in different directions with inhomogeneous amplitude induced by heterogeneous distribution of cardiac muscle fibers in nature^25^. Therefore, the output voltage obviously depends on the device suture position and orientation of the device. To better understand this we conducted energy harvesting test in open chest with the UFEH sewed in different locations on the heart of the swine, trying to find the most effective location with maximum and stable output voltage for energy harvesting. Seven different places have been tested, and results indicate that the output signal closely related to the location and the orientation of the device mounted to the heart. Here we take the comparison between two locations to illustrate the correlation of location and output voltage. In Fig. 3a, the device was fixed in between the left ventricular (LV) apex and right ventricle (RV) perpendicular to the left descending coronary artery, i.e., the apical anterior ventricular wall, which belongs to segment 13 according to the Standardized Myocardial Segmentation and Nomenclature of the American Heart Association^26^. Figure 3b shows the corresponding output signal with a peak-to-peak voltage of as high as 3 V. In Fig. 3c, the UFEH was between the anterior atrioventricular groove and the right ventricular outflow tract parallel to the left anterior descending coronary artery and perpendicular to the orientation in Fig. 3a. The corresponding output voltage is shown in Fig. 3d. However, the peak-to-peak voltage can only reach 0.8 V, 73.3% smaller than the above. Obviously, the peak-to-peak output voltage heavily relies on the cardiac segment to which the device is fixed owing to the complicated motions of heart. Among these seven locations, we have found that the apical anterior ventricular wall (the region between left ventricular apex and right ventricle), i.e., cardiac segment 13, is the optimal and most effective location to generate maximal peak-to-peak voltage. As we all know the contraction of the ventricles is stronger than the other two atria. The mechanical function of the LV is to pump oxygenated blood into aorta and leading to the systemic circulation while that of the RV is to pump the deoxygenated blood through pulmonary arteries leading to the lung. The maximum strain of LV in systole can reach about 35% while both anterior and posterior walls become thickening (i.e. expansion)^27^,^28^,^29^. The deformation of other part (e.g. RV) is much smaller than that of LV. In fact, of all the segments in normal hearts^26^, segment 13 is the location where maximal epicardial deformation occurs, which can be seen in the setting of routine echocardiographic evaluations and is associated with the “wrinkles” in the epicardium of the right ventricle observed intraoperatively after weaning from cardiopulmonary bypass. We are speculating from anatomic and physiological considerations that segment 13 represents the mid-chamber portion of the left ventricle, which would be associated with the greatest contribution to the stroke volume. Thus, it is associated with the greatest epicardial deformation, which makes this suture location optimal for energy harvesting based on piezoelectricity.

**Figure 4:**
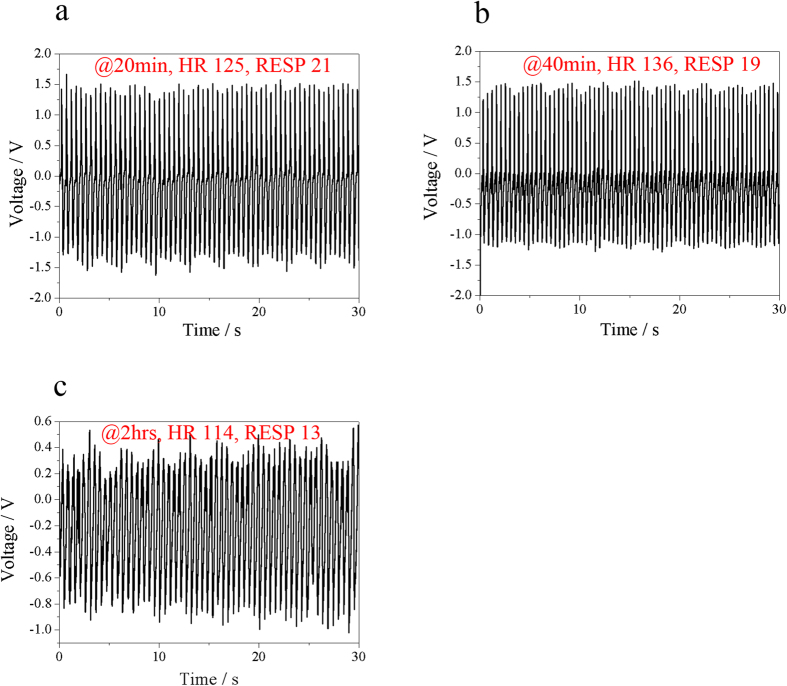
Output voltage of UFEH depends on the status of heart (e.g. strong or weak heart). (**a**) Output voltage at 20 min after chest is open. (**b**) Output voltage at 40 min after chest is open. (**c**) Output voltage at 2 hours after chest is open.

Following these findings, we proceed to study the UFEH’s performance under different functional status of the heart and lungs, since cardiac contractility alters in various physiological and pathological settings^30^. The easiest way is to keep track of the changes in output voltage over time while the UFEH remains on segment 13. Figure 4 indicates that under adequate anesthetic control, the longer time the testing lasts, the weaker the heart motion as reflected by the values of hemodynamic parameters and physiological index recorded by the medical monitors, such as respiration rate (RESP) and heart rate (HR). Peak-to-peak voltage is 3 V at 20 minutes after start of testing with a blood pressure of 76 mmHg, and heart rate and respiration rate is 125 bpm and 21 bpm respectively 20 min, shown in Fig. 4a. Obviously, as the time elapses, the motion of heart becomes weak and the heart rate and respiration rate drops to 114 bmp and 13 bmp after 2 hours. Correspondingly, the peak-to-peak voltage decreases by 10% to 2.7 V at 40 minutes (shown in Fig. 4b), and drops by 56% in magnitude to 1.3 V at 2 hours (shown in Fig. 4c). This correlation between heart status and output voltage of the UFEH indicates that the energy harvesting efficiency is dependent on the subject also, which means rather than setting a uniform standard only on theoretical analysis, a model with statistics in heart strength of an individual is required. An estimation of the heart motion intensity can be achieved with all kinds of medical image technology. And this model needs further research to bring energy conversion of mechanical energy of heart motion into electric closer to clinical application.

**Figure 5:**
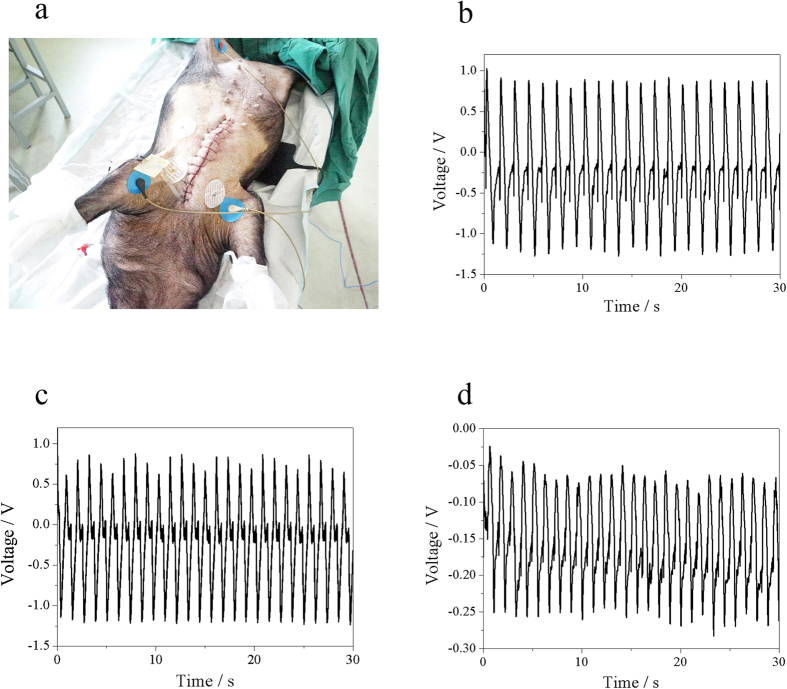
Signal from open chest, close chest and awake settings. (**a**) Image of close chest measurement. (**b**) Output voltage in chest open setting. (**c**) Output voltage in chest closed setting. (**d**) Output voltage when the subject is relieved from the anesthesia (chest remains closed).

In order to further evaluate the viability and benefit of the UFEH *in vivo* over time, we carried out the test under two more settings, i.e., energy harvesting after closure of the chest as well as after recovery from anesthesia, which is closer to normal circumstances than the setting of an open chest. Figure 5a shows the swine recovers from the anesthetic after closing the chest supported by life maintenance system. The flexible ACF cable was sewed through the muscle and enabled the output voltage to be measured. The video in the Supplementary Information (Closing the chest & Recovering from anesthesia) shows the whole surgery process and legs of the experimental swine move after recovering from anesthesia to demonstrate the conscious condition. Figure 5b–d show the output voltage obtained from open-chest, close-chest and conscious situation respectively. Analysis of the output signal shows that in all three settings, periodically steady and uniform output voltage synchronous with the cardiac cycle is harvested, and the peak-to-peak voltage is 2.3 V, 2.2 V and 0.3 V respectively. The output voltage is largest with an opened chest, abates a little bit after chest closure and decreases further after the animals awake from anesthesia. It is probable that the magnitude of the energy harvester deformation is limited by the increase of intrathoracic pressure and compression from adjacent tissues after chest closure, as well as the increase in cardiac afterload after anesthetic recovery. On the contrary, in the chest open situation the motion of the heart is not constrained in the open air so that the deformation amplitude is larger than that in the other two situations, so as the output voltage. The output voltage of the awake setting is far smaller than that of open-chest, because the swine is suffering when cardiac function may be affected by the anesthesia and surgical injury from thoracotomy, pericardiotomy and sutures, which is confirmed by the decreased values of monitored hemodynamic and physiological index. However the output signal is still periodically steady and regular, indicating that the device is still working properly without destruction or degeneration, and the decreased output voltage implies the reduced amplitude of the heart motion. It is desirable that the flexible PZT energy harvester can generate a peak-to-peak voltage of 2–3 V in practical usage, when it is implanted within closed chest.

The suture manner for the energy harvester to be mounted onto the heart also has effect on the output signal because it constrains the deformation of the piezoelectric part of the device, which directly affects the generated electric energy through piezoelectric effect. During *in vivo* test we’ve tried 3 ways to sew the UFEH onto the heart, i.e. one stitch, two or four stitches at both ends of the device at the same location on the heart, and found that putting four-stitch at each end results in the highest output voltage with better stability. Therefore we suggest the energy harvester be mounted with four stitches at each end to acquire the highest efficiency of energy transformation, due to formation of the clamped constrains at both ends of the device and decrease of the unsupported length, which enable the PZT stacks to deform largely screening unnecessary twisting and rotating.

## Discussion

So far the ability of the PZT-based UFEH to convert biomechanical energy into electricity has been demonstrated and studied, and the output voltage can be as high as 3 V, which is approximate to the biomedical implants’ power requirement. For instance, the working voltage of commercial lithium-iodine battery for cardiac pacemaker is 2.4 ~ 2.8 V. There are three ways to modulate the output voltage of the UFEH to accommodate various demands of different implantable devices, including 1) integration of the UFEH with the heart with different locations or mounting methods, as this study shows that different locations or manners of mounting are associated with the varying output voltages; 2) modification of the PZT stacks’ alignment for different output voltages, with an increased number of arrays in series leading to an rise of the power to supply, and vice versa; 3) use of several flexible energy harvesters on the heart in series to get even larger output voltage to support multiple implantable devices if necessary, or a single device with large power demand, with the total output voltage conforming to rule of linear superposition.

The PZT-based UFEH is designed and fabricated by transfer-printing to convert biomechanical energy of heart motion into electricity as a possible substitution of power supply for medical implants. *In vivo* test of energy harvesting is conducted in swine models in the settings of an opened chest, closed chest and anesthetic recovery to demonstrate the feasibility and benefits of the UFEH, and influence of mounting locations and suture manners on the output signal are also investigated. Results show that the device proposed here barely brings no extra burden or damage to the heart due to its ultra-flexibility and light weight. The peak-to-peak voltage can reach as high as 3 V when the UFEH is fixed at segment 13 (between the left ventricular apex and the right ventricle) after chest closure, which is sufficient for the power requirement of commercially available pacemakers.

## Methods

### Fabrication of UFEH for Biomechanical Energy Harvesting

The whole process can be divided into (a) film deposition and formation of PZT stacks structure; (b) transfer printing PZT stacks to soft substrate with the thickness of 75 um and interconnecting each PZT stack with gold interconnects; (c) connecting the device with flexible ACF cable.

The manufacture starts with sequential film deposition of SiO_2_, Ti, Pt, PZT Cr and Au on the silicon wafer with the thickness of 600 nm, 20 nm, 300 nm, 500 nm, 10 nm and 150 nm respectively, in which the PZT is deposited by sol-gel spin casting method (500 nm, MEMS). Besides the materials introduced in the design, SiO_2_ plays the role of sacrificial layer for releasing the whole device from the silicon wafer after patterning, and Ti acts as the bonding layer between the bottom electrode Pt and sacrificial layer SiO_2_ to keep the whole structure stuck to the substrate during nano-/micro- fabrication. The functional part of the energy harvester including top and bottom electrode as well as PZT ribbons is patterned into arranged array by 3 steps of lithography and 4 steps of wet etching with different masks to make sure the size of the upper layer is smaller than lower ones in each stack, which guarantees the exclusion of breaking down in the PZT capacitor stack. In order to improve the performance of the energy harvester, it is polarized by DC power supply for more than one hour under the electric field of over 10 MV/m, where the time and electric field intensity should be carefully handled and adjusted to prevent the device from breaking down during the polarization process.

### *In vivo* Testing

All *in vivo* experiments performed were approved by the Ethics Committee (Ethics Committee of Beijing Anzhen Hospital, Capital University of Medical Sciences) and in accordance with relevant guidelines and regulations. Experimental swine raised especially for cardiovascular research are selected for *in vivo* testing. Food for the selected swine is prohibited 12 hours before *in vivo* test to prevent vomit during the experiment. After introduction of intravenous anesthesia, endotracheal intubation and mechanical ventilation are initiated to control the airway. Blood pressure, heart rate, pulse and respiration are measured and monitored continuously, which helps evaluate the animals’ physiological status during the test. After the swine chest is opened, the ultra-flexible energy harvester is mounted to the epicardium by surgical sutures with particular care to avoid injury to the coronary arteries. AD/DA card (Art-control USB2815) is used to capture the signal generated during energy harvesting and transmit it to a computer for display and analysis. The resistance R of the voltmeter is positively correlated with the measured voltage as is expressed in equation 1, in our case the resistance of the AD/DA card is 10.8 MΩ.

### Comparison of Bending Stiffness between Medical UFEH and Dacron Patches

The bending stiffness of a film with unit width is defined as 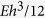, in which 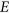 is elastic moduli and h is the thickness of the film. Due to the fact that thickness and covering area of the Kapton substrate are much larger than others, Kapton contributes most to the bending stiffness of UFEH. Therefore, the ratio of bending stiffness of the UFEH to Dacron patch, a widely used surgical material, is 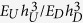, where the subscript U stands for UFEH, and D stands for Dacron. For a commonly used Dacron (Mersilene), 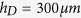, 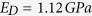
24, and that of the UFEH is 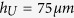, 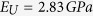, which gives the ratio 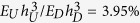 meaning the bending stiffness of the UFEH is smaller than that of Dacron. Both theoretical and experiment performances show that the bending stiffness of the UFEH is comparable to or even smaller than the commercially available Dacron patches which is widely used in various cardiac operations. The mounting of UFEH on the heart for energy harvesting would not bring any extra damage or burden to the heart with careful sutures and proper manipulations.

## Additional Information

**How to cite this article**: Lu, B. *et al.* Ultra-flexible Piezoelectric Devices Integrated with Heart to Harvest the Biomechanical Energy. *Sci. Rep.* **5**, 16065; doi: 10.1038/srep16065 (2015).

## Supplementary Material

Supplementary Video 1

Supplementary Video 2

Supplementary Video 3

Supplementary Video 4
